# Sodium Oxybate (SMO) as Part of Agonist Opioid Treatment in Alcohol–Heroin-Addicted Patients

**DOI:** 10.3390/jcm14124016

**Published:** 2025-06-06

**Authors:** Angelo G. I. Maremmani, Filippo Della Rocca, Matteo Pacini, Silvia Bacciardi, Silvia Cimino, Luca Cerniglia, Mario Miccoli, Icro Maremmani

**Affiliations:** 1 VP Dole Research Group, PISA-School of Addiction Medicine, G. De Lisio Institute of Behavioural Sciences, Via di Pratale 3, 56121 Pisa, Italy; angelogiovanniicro.maremmani@unicamillus.org (A.G.I.M.); filippo.dellarocca@yahoo.it (F.D.R.); studiompacini@gmail.com (M.P.); s.baciard@gmail.com (S.B.); 2 Addiction Research Methods Institute, World Federation for the Treatment of Opioid Dependence, 225 Varick Street, Suite 402, New York, NY 10014, USA; mario.miccoli2020@virgilio.it; 3 UniCamillus, International Medical University in Rome, Via di Sant’Alessandro, 8, 00131 Rome, Italy; 4 Addiction Unit, Department of Mental Health and Addictions, ASL5 Liguria NHS, Via Dalmazia 1, 19124 La Spezia, Italy; 5 Department of Psychiatry and Addictions, Section of Psychiatry, North-Western Tuscany Local Health Unit, Tuscany NHS, Versilia Zone, Via Aurelia 335, 55041 Lido di Camaiore, Italy; 6 Department of Clinical, Dynamic and Health Psychology, Sapienza University, 00185 Rome, Italy; silvia.cimino@uniroma1.it; 7 Faculty of Psychology, International Telematic University Uninettuno, 00186 Rome, Italy; l.cerniglia@uninettunouniversity.net; 8 Department of Human and Social Sciences, Mercatorum University, Piazza Mattei, 10, 00186 Roma, Italy

**Keywords:** alcohol use disorder, heroin use disorder, HUD, sodium oxybate, SMO, agonist opioid treatment, OAT, long-term treatment, retention in treatment, efficacy, safety

## Abstract

**Background:** Alcohol use disorder in the context of heroin addiction presents a significant challenge for clinicians, particularly in selecting the most appropriate pharmacological treatment. **Methods:** The present study aimed to retrospectively evaluate the efficacy of a six-month methadone maintenance (MM)/sodium oxybate (SMO) combination treatment in reducing ethanol intake among chronic alcohol-dependent patients with heroin use disorder (HUD). Specifically, we compared outcomes between those who continued SMO treatment after alcohol detoxification (MM/SMO-Maintained) and those who discontinued it (MM/SMO-Detoxified). Data were recruited using the ‘Pisa Addiction Database’ through a retrospective, naturalistic, cross-sectional comparative design involving a single patient assessment. **Results:** Our results indicate that treatment retention was higher in the MM/SMO-Maintained group. Conversely, discontinuing SMO treatment after alcohol detoxification was associated with a higher likelihood of dropout. At the endpoint, the MM/SMO-Maintained group showed significant improvement and was considered less severely ill. **Conclusions:** Long-term SMO treatment has proven to be well tolerated and effective in preventing relapse in individuals with both alcohol and HUD undergoing agonist opioid treatment. SMO may be considered the closest pharmacological option to substitution therapy for alcohol use disorder, and ongoing agonist opioid treatment should not preclude its co-administration.

## 1. Introduction

Substance use disorders (SUDs) present significant challenges not only for individuals but also for public health systems. Historically, research has focused on substance-specific addictions and corresponding treatment strategies. However, in clinical practice, the prevalence of patients with isolated substance use—such as heroin use disorder (HUD), cocaine use disorder, or alcohol use disorder (AUD)—is increasingly rare [1,2,3]. Instead, polysubstance use has become more common, leading to complex clinical presentations and making treatment more difficult [4,5]. Polysubstance use refers to the consumption of multiple substances either concurrently or sequentially over time [6]. This pattern is associated with lower treatment efficacy, higher dropout rates [7,8], and poorer therapeutic response [9,10,11]. Alcohol use, in particular, frequently co-occurs with other SUDs. Numerous studies have confirmed the association between alcohol and opioids, including heroin [12,13,14]. The concurrent use of these substances is partly due to their shared sedative effects and cross-tolerance. In one study, up to 40% of individuals with HUD also used alcohol [15]. Other sources report rates between 50% and 75%, with approximately 10% of patients experiencing alcohol-related physical complications [12]. The lifetime and current prevalence of AUD in HUD populations is estimated at 15–35% [13] and AUD is present in 10–20% of individuals who use street drugs and up to 30% of patients in methadone maintenance treatment (MMT) programs [12,16].

While opioid agonist treatments represent the gold standard for HUD—due to proven efficacy and safety [17,18]—the same cannot be said for AUD. Currently, disulfiram, acamprosate, and naltrexone are the approved medications for maintaining abstinence in AUD. However, many patients fail to respond to these treatments [19], highlighting the need for additional pharmacological options [20]. Sodium oxybate (SMO), an oral solution, has been approved in Italy and Austria since 1991 and 1999, respectively, for both alcohol withdrawal and relapse prevention [21,22]. SMO is the sodium salt of gamma-hydroxybutyric acid (GHB), a naturally occurring short-chain fatty acid structurally related to gamma-aminobutyric acid (GABA). GHB acts as both a precursor and metabolite of GABA, binding with high affinity to GHB-specific receptors and with low affinity to GABA-B receptors, which are widely expressed in brain regions involved in reward, stress, and motor control. SMO replicates these effects in a controlled and clinically predictable manner. Its therapeutic efficacy in AUD is believed to derive from modulation of GABAergic transmission, inhibition of glutamate release, and normalization of the disrupted stress–reward circuitry [23,24,25,26,27]. Additionally, SMO enhances slow-wave sleep and transiently reduces dopamine release in the nucleus accumbens, followed by rebound dopaminergic activation. This biphasic modulation may help stabilize reward systems in individuals with alcohol dependence. While pharmacologically distinct, alcohol and SMO share central nervous system (CNS) depressant properties and exhibit cross-tolerance. SMO can mimic the anxiolytic and sedative effects of alcohol, making it particularly effective in patients with high levels of alcohol craving. Unlike alcohol, however, SMO does not generate neurotoxic metabolites and has a more predictable safety profile [28,29,30]. The anti-craving effect of SMO, though different in mechanism, may resemble that of methadone or buprenorphine in HUD. It is currently considered the closest available pharmacological option to a substitution therapy for AUD [31].

The relationship between SMO and the opioid system is still not fully understood. Some studies have demonstrated that SMO can alleviate withdrawal symptoms in individuals who discontinued methadone abruptly, with good tolerability over short treatment periods [32,33]. Interestingly, several authors have observed increased alcohol consumption during MMT. This behavior may represent an attempt to bypass methadone’s stabilizing effects on the opioid system. When properly dosed, methadone reduces dopaminergic hypersensitivity and normalizes opioid tone. Some patients, however, may perceive this stabilization as a reduction in emotional or reward-related responsiveness. In such cases, alcohol—a cross-tolerant CNS depressant—may be used to induce disinhibition or euphoria, undermining methadone’s therapeutic effect [12,16,34]. A compensatory pattern has been described in which alcohol use rises as street opioid use declines and vice versa. This “see-saw” dynamic may reflect a form of addiction switching or emotional self-regulation in patients with incomplete opioid stabilization. In individuals who discontinue MMT or receive subtherapeutic doses, alcohol use may intensify to compensate for residual opioid dysregulation [12].

When alcohol use persists despite adequate MMT, based on the Dole and Nyswander methodology [35,36], this may indicate a dual disorder—where both HUD and AUD coexist independently [1,13,37,38,39,40,41,42]. In such cases, increasing methadone dosage may no longer be effective and a second anti-craving agent may be required. While MMT has robust efficacy and safety data, no similarly validated maintenance therapy exists for AUD. SMO may represent the best current option in this regard.

In the present study, we hypothesized that maintaining SMO after alcohol detoxification in patients undergoing MMT (MM/SMO-Maintained) would result in better treatment retention compared to patients who discontinued SMO after detoxification (MM/SMO-Detoxified). SMO is known to be effective both in alcohol withdrawal and in supporting abstinence [27,43,44,45].

This retrospective study evaluated the six-month efficacy of combined MMT and SMO treatment in reducing alcohol consumption among chronic AUD patients with HUD. We compared outcomes between the MM/SMO-Maintained and MM/SMO-Detoxified groups and analyzed baseline demographic and clinical predictors of treatment response.

## 2. Materials and Methods

### 2.1. Design of the Study

This study is based on data extracted from the Pisa Addiction Database, a repository developed within the framework of previous research on MMT Programs in Italy, as described in earlier publications. The database includes anonymized individual-level data collected either during prior research protocols or as part of routine clinical management. Patients not enrolled in a specific research protocol were included in the database based on two main criteria: (1) a request to receive treatment, and (2) willingness to participate in future research surveys. Participation in the database was entirely voluntary and did not influence access to standard care. Patients could withdraw their consent at any time without providing justification. All studies contributing data to the Pisa Addiction Database were previously approved by the Ethics Committee of the University of Pisa. Therefore, no additional ethical approval was required for this secondary analysis.

The study followed a retrospective, naturalistic, cross-sectional comparative design. Data were obtained from a single clinical assessment with the aim of evaluating differences in clinical severity and outcome variables between two groups: the MM/SMO-Maintained and MM/SMO-Detoxified groups.

### 2.2. Sample

**Figure 1:**
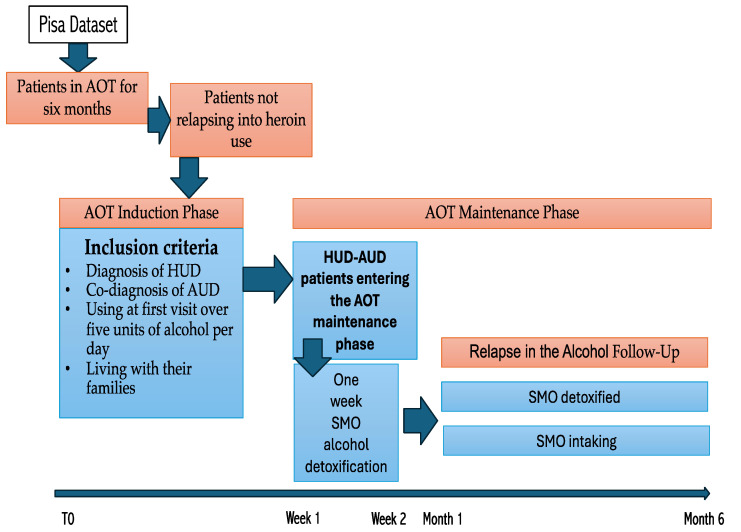
Study planning. Note: AOT = agonist opioid treatment; HUD = heroin use disorder; AUD = alcohol use disorder; SMO = sodium oxybate.

We considered all cases recorded in the Pisa Addiction Database involving patients treated with methadone and SMO for up to six months at the V.P. Dole Dual Disorder Unit, Santa Chiara University Hospital, Pisa (Italy), over a ten-year period (2010–2020). The study included individuals diagnosed with HUD and co-occurring AUD, as per Diagnostic Statistical Manual (DSM) criteria [46,47], without any lifetime psychiatric comorbidities. At the time of their initial evaluation, these patients were consuming more than five alcoholic drinks per day and resided with their families. All participants had available follow-up data for a six-month period. We excluded individuals with severe hepatic impairment, chronic medical conditions, recent engagement in structured psychotherapy (within the past year), or any lifetime psychiatric diagnoses. A total of 21 patients who received combined methadone–SMO treatment (MM/SMO-Maintained) were included. These individuals were matched, as closely as possible by age and sex, with 21 patients who underwent alcohol detoxification with SMO but discontinued its use after detoxification (MM/SMO-Detoxified group) (Figure 1).

The final sample included 42 patients (mean age 40 ± 11 years; 20 males). The majority were female (52.4%), in a stable relationship (64.3%), blue collar—employed in manual labor occupations such as construction, manufacturing, or maintenance work (64.3%)—and had a low level of formal education (66.7%). Most participants reported adequate financial resources (88.1%) and were not living alone (76.2%).

Among them, 21 patients underwent methadone maintenance (MM) with alcohol detoxification using SMO (MM/SMO-Detoxified), while 21 patients continued with MM and SMO co-maintenance (MM/SMO-Maintained). The two groups were well balanced in terms of baseline characteristics, including age, sex, education level, marital status, employment status, income, and living arrangement. Detailed demographic data are presented in Table 1.

### 2.3. Assessment

Alcohol intake was quantified in terms of alcohol units. Daily consumption was assessed based on the number of standard drinks reported by each patient. One unit of alcohol was defined as approximately 12 g of pure ethanol, corresponding to a typical glass of wine (125 mL), a can of beer (330 mL), or a shot of spirits such as whiskey (40 mL). Abstinence from alcohol was monitored through reports from family members, one or two of whom were designated to observe the patient’s alcohol use and medication adherence (methadone and SMO). A positive outcome (responder) was defined as complete abstinence from alcohol during the follow-up period. Non-responders included patients who refused to continue treatment, voluntarily dropped out of the program, or relapsed into alcohol use. Clinical status was evaluated using the Clinical Global Impressions (CGI) scale [48], administered by a clinician blinded to the patient’s group allocation and alcohol consumption data. Blinding was implemented to minimize bias, as the CGI is intended to reflect global psychological, social, and occupational functioning without being influenced by substance use variables. The CGI includes three global measures: Severity of Illness, Global Improvement, and the Efficacy Index. The first two are rated on a 7-point scale. The Efficacy Index combines therapeutic benefit with side-effect burden, calculated as a ratio between therapeutic effectiveness (rated from 1 = Unchanged or Worse to 4 = Marked) and adverse effects (rated from 1 = None to 4 = Outweighs). A higher ratio indicates greater net benefit. Details of the CGI scoring system are summarized in Table 2.

### 2.4. Procedure

At baseline, all patients initiated MMT and SMO treatment. MMT was delivered according to the Dole and Nyswander methodology. Since 1993, the Pisa-MM program has adopted a high-threshold clinical protocol for opioid addiction, with a strong emphasis on pharmacological stabilization and long-term maintenance. Following induction, methadone doses were progressively increased until patients reached stabilization, defined as having no more than one positive urine drug screen for illicit opioids, cocaine, or benzodiazepines within a 60-day period. The methadone dose achieving this outcome was designated as the “stabilization dose”. There was no predetermined upper limit for methadone dosage. However, patients who failed to reach stabilization within one year were discharged and referred to local addiction services. Dose adjustments were made based on toxicological monitoring; improvements in social functioning alone were not sufficient to maintain dose levels if urinalyses remained positive. Dose self-adjustment by patients was not permitted. Once patients demonstrated full compliance with program requirements, take-home doses were allowed for up to seven days. The SMO protocol followed a three-phase structure. During the first week (Phase I), patients received SMO at 50 mg/kg/day, divided into three doses every four hours, to manage alcohol withdrawal and promote abstinence. In Phase II, applicable only to the MM/SMO-Maintained group, the dose was titrated up to 100 mg/kg/day based on individual clinical response. Due to SMO’s short half-life [49,50,51], the total daily dose was administered either in three doses every four hours or six doses every 2.5 h [52]. Dose self-adjustment was permitted, but only with medical approval. Phase III consisted of six months of maintenance treatment. In contrast, patients in the MM/SMO-Detoxified group discontinued SMO after the initial detoxification week but continued MMT for the remainder of the six-month follow-up. No structured psychotherapy was provided, and the number of visits was not standardized. In general, patients were seen daily during the first week and then at least twice per month. All participants provided written informed consent before initiating any pharmacological treatment. As the service operated under routine clinical care conditions, there was no randomization or deviation from standard procedures. Patients were expected to actively participate in their treatment plans, attend scheduled appointments, meet regularly with physicians and case managers, and engage in group activities when clinically indicated. All treating physicians were psychiatrists with at least two years of specialized training in addiction medicine. Data were recorded anonymously, and participants could not be identified in any publication or report.

### 2.5. Data Analysis

Retention in treatment was assessed using survival analysis. Kaplan–Meier survival curves were generated, and group differences were evaluated using the Wilcoxon (Breslow) test. Censored observations included patients who were still in treatment at the study endpoint or those who left the program for reasons unrelated to treatment outcomes (e.g., transfer to another facility or incarceration for prior offenses). Treatment dropout or expulsion was considered a terminal event. Positive outcomes were defined as either: (1) successful transfer to another service following a period of stabilization or completion of maintenance treatment, or (2) continued engagement in treatment at the endpoint, with clinical stabilization. Negative outcomes included failure to achieve stabilization within one year or relapse into addictive behaviors following a period of stabilization. To assess the association between treatment modality (MM/SMO-Detoxified vs. MM/SMO-Maintained) and retention, a Cox proportional hazards regression model was used. Sociodemographic and clinical variables (age, sex, baseline severity of illness) were included as potential confounders. Differences between groups on Clinical Global Impressions (CGI) scores at baseline and at endpoint were evaluated using the Mann–Whitney U test, given the ordinal nature of the scale. All analyses were performed using SPSS software, version 25.0.

## 3. Results

### 3.1. Retention in Treatment

**Figure 2:**
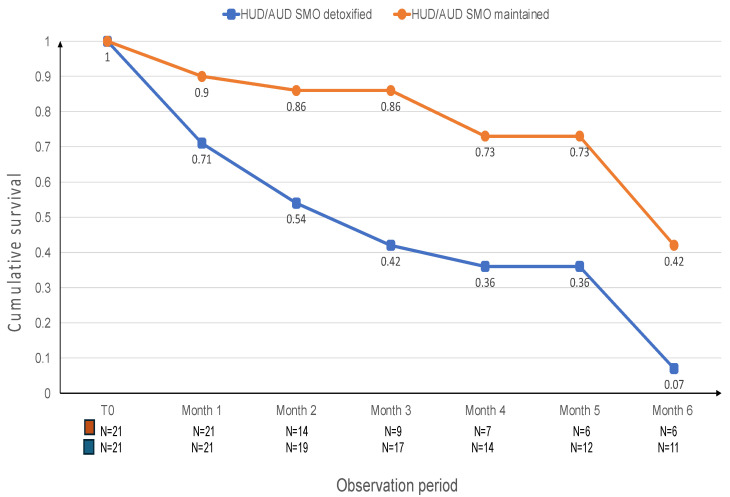
Alcohol relapsing behavior in AOT responder HUD/AUD patients according to the use of SMO as co-maintenance treatment. Legend: HUD/AUD = heroin use disorder/alcohol use disorder; SMO = sodium oxybate.

Retention was assessed monthly over a six-month observation period. At one month, all 42 patients (21 per group) remained in treatment. By month two, 14 patients in the MM/SMO-Maintained group and 19 in the MM/SMO-Detoxified group were still engaged. The numbers progressively declined: at month three, 9 (Maintained) vs. 17 (Detoxified); month four, 7 vs. 14; month five, 6 vs. 12; and at month six, 6 vs. 11, respectively. The median survival time was 100.9 days for MM/SMO-Maintained patients and 180.0 days for MM/SMO-Detoxified patients. Relapse into alcohol use occurred more frequently in the MM/SMO-Maintained group. During the first month, 6 Maintained patients (29%) and 2 Detoxified patients (10%) relapsed. In month two, the relapse rates were 23% vs. 5%; in month three, 22% vs. 0%; in month four, 14% in both groups; no relapses were recorded during month five; and in month six, relapse occurred in 4 Maintained patients (80%) and 3 Detoxified patients (43%). At month five, 13 MM/SMO-Maintained and 5 MM/SMO-Detoxified patients were still in treatment or had exited the study for reasons unrelated to alcohol relapse. The cumulative survival proportion at six months was 0.42 for the MM/SMO-Maintained group and 0.07 for the MM/SMO-Detoxified group. This difference was statistically significant (Wilcoxon statistic = 8.45, *p* = 0.004) (see Figure 2).

### 3.2. Predictors of Terminal Events

Table 3 presents the correlation between treatment outcome and key variables, including sex, age, baseline severity of illness, and type of treatment received. Among these, only the treatment modality showed a statistically significant association with dropout rates. Specifically, patients who underwent alcohol detoxification and discontinued SMO were more likely to discontinue treatment, indicating a negative correlation between cessation of SMO and treatment retention.

### 3.3. Clinical Global Index

At baseline, 10 patients (47.6%) in the MM/SMO-Detoxified group were rated as markedly ill, 10 (47.6%) as severely ill, and 1 (4.8%) as extremely ill. In the MM/SMO-Maintained group, 9 patients (42.9%) were rated as markedly ill and 12 (57.1%) as severely ill. These differences were not statistically significant according to the Mann–Whitney U test (z = 0.12; *p* = 0.897). At the six-month endpoint, 27 patients showed clinical improvement, 15 showed no meaningful change, and none experienced a worsening of illness severity. The change in illness severity over time was statistically significant (z = −4.60; *p* < 0.001). Importantly, patients in the MM/SMO-Maintained group exhibited significantly better scores across all three dimensions of the Clinical Global Impressions (CGI) scale compared to the MM/SMO-Detoxified group (see Table 4).

## 4. Discussion

This study aimed to retrospectively evaluate the six-month efficacy of combined methadone maintenance/sodium oxybate (MM/SMO) treatment in patients with heroin and alcohol use disorders. Our findings indicate that treatment retention was significantly higher among patients who continued SMO following alcohol detoxification (MM/SMO-Maintained), compared to those who discontinued SMO after detoxification (MM/SMO-Detoxified). Treatment dropout was associated exclusively with treatment type, suggesting that the discontinuation of SMO increased the likelihood of dropout. At endpoint, MM/SMO-Maintained patients also demonstrated greater clinical improvement and were rated as less severely ill than their detoxified counterparts.

It is important to contextualize these results within the framework of opioid agonist therapy. All patients in this study were treated according to the Dole and Nyswander methodology [36,53], which emphasizes individualized dosing and a strong focus on the maintenance phase. Adequate methadone dosing must exert both blocking and anti-craving effects to fully stabilize the opioid system [13,37,39,54,55,56,57]. In patients with polysubstance use—particularly those using alcohol or benzodiazepines in addition to heroin—failure to achieve this stabilization may lead to ongoing substance use despite treatment. This clinical presentation has been described as “masked heroinism” [38], a form of alcohol use that arises secondarily as compensation for an underdosed or suboptimally managed opioid dependence. Caputo and colleagues demonstrated that even short-term methadone treatment could significantly reduce alcohol consumption in heroin-addicted individuals without a formal AUD diagnosis [58].

Unlike opioid use disorder (OUD), for which methadone and buprenorphine are well-established maintenance treatments, there is no pharmacological agent for AUD that offers comparable efficacy. SMO represents one of the most promising candidates to fill this gap. Several randomized controlled trials (RCTs) have shown its effectiveness in achieving and maintaining alcohol abstinence [19,22,59,60]. These findings have been reinforced by large-scale trials such as the one conducted by Guiraud et al. [27], which confirmed the sustainability of SMO’s effects even after discontinuation. Importantly, most of these studies were conducted in individuals with “pure” AUD, and excluded those with co-occurring SUDs. This makes our findings particularly relevant, as they offer preliminary real-world evidence of SMO’s utility in a population with more than a single addiction. The efficacy of SMO has been demonstrated with large effect sizes in patients with treatment-resistant AUD [61], as well as in RCTs involving high-severity populations—namely, those characterized by low placebo response rates [19]. Additionally, findings from a Phase IIb double-blind, randomized, placebo-controlled trial investigating a novel abuse- and misuse-deterrent formulation of SMO showed significant improvements in this severe clinical subgroup, both in terms of percentage of abstinent days and overall abstinence rates [62]. Collectively, these results support the effectiveness of SMO in managing alcohol withdrawal syndrome and in maintaining long-term abstinence from alcohol [21,63,64].

It is important to note that most studies evaluating SMO have focused on individuals with AUD as a singular diagnosis. However, as previously discussed, the prevalence of isolated substance use disorders has markedly declined in clinical practice. The safety and efficacy of SMO in the context of polysubstance use and complex pharmacological regimens remain areas of ongoing debate. A key question among clinicians is whether the co-administration of SMO with opioid agonist treatments can be considered safe and clinically appropriate.

Several safety-related considerations must be addressed. First, available evidence does not indicate significant pharmacokinetic or pharmacodynamic interactions between SMO and methadone when both are administered under appropriate medical supervision. However, due to their shared depressant effects on the CNS, co-administration can increase the risk of excessive sedation or respiratory depression. For this reason, careful dose titration and close clinical monitoring are essential, particularly during the induction phase and when dose adjustments are made [65].

Second, the potential for misuse must be clarified. SMO is the sodium salt of GHB, a substance known for its abuse potential in recreational settings. However, its therapeutic use, when prescribed and supervised by healthcare professionals [43], should not be equated with illicit GHB consumption. Street names for recreational GHB include “Georgia Home Boy”, “Juice”, “Liquid Ecstasy”, “G”, and “Fantasy” [66,67]. Unlike SMO, illicit GHB is often used without dosing control, which significantly increases the risk of intoxication, dependence, and criminal misuse. Polysubstance use, especially the combination of ethanol with CNS depressants such as opioids or GHB, is known to increase the likelihood of adverse outcomes, including medical emergencies and hospitalizations [68]. Nonetheless, clinical and epidemiological data indicate that the risk of non-medical use of SMO is lower than previously feared by many clinicians [31]. SMO has demonstrated good tolerability in both randomized trials and routine clinical practice, particularly in Italy and Austria [45]. Cases of non-medical use, dependence, and misuse have been reported almost exclusively in association with illicit GHB, not with prescribed SMO [69,70]. Notably, a Cochrane meta-analysis [22] and other studies [52,71,72] confirm that therapeutic doses of SMO do not lead to the development of tolerance or withdrawal syndromes, either when used for alcohol withdrawal or for relapse prevention.

When used in a controlled clinical context, SMO appears to have a low risk of misuse, particularly among patients receiving opioid maintenance therapy. Several studies, including those cited in the present manuscript, suggest that the incidence of craving for or abuse of SMO is limited (approximately 10–15%), primarily in individuals with psychiatric comorbidities or a prior history of cocaine or heroin dependence [21,52,73]. This observation may be explained by dopaminergic dysregulation system [74]. Patients with chronic exposure to heroin or cocaine often exhibit downregulation of D1 and D2 receptors, which may make them more sensitive to the rewarding effects of SMO at high doses (typically >50 mg/kg/day) [75,76]. Therefore, in individuals with complete remission from heroin or cocaine use, initiating SMO should be approached cautiously. However, these risks should not discourage physicians from prescribing SMO to patients with AUD who are concurrently receiving methadone, particularly when the latter is administered according to the Dole and Nyswander model [73]. In fact, patients stabilized on opioid maintenance therapy may be less vulnerable to SMO-related craving, possibly due to the normalization of the reward and endorphin systems achieved by long-term opioid agonist treatment. Our own experience at the V.P. Dole Dual Disorder Unit supports this view. More than 15 years ago, we described a case series of 13 polysubstance users in which SMO showed some degree of effectiveness in reducing alcohol use, despite limitations in adherence and completeness of response [77]. As highlighted throughout this discussion, SMO remains one of the most promising pharmacological options for the treatment of severe AUD in maintenance settings. Even in the context of polysubstance use, SMO may serve as an effective component of relapse prevention strategies. Accordingly, the presence of opioid agonist therapy (e.g., methadone or buprenorphine) should not automatically preclude SMO co-administration [78].

Regarding contraindications, SMO should not be prescribed to patients with a history of GHB abuse, untreated epilepsy, or active dependence on other sedatives unless a comprehensive treatment plan and appropriate medical supervision are in place. It should also be avoided in patients unable to comply with monitoring protocols. Additional caution is recommended in individuals with respiratory insufficiency or sleep apnea [25,79].

Clinical Implications:

The findings from this study have several practical implications for the treatment of patients with HUD and AUD. Based on our results and clinical experience, we recommend the following:

- Supervised administration: Self-administered doses of SMO should be monitored by a caregiver or healthcare provider to minimize risk.
- Use of lower single doses: Particularly in HUD patients, as higher doses may be more easily distinguishable and potentially reinforcing [75].
- Monitoring of subjective effects: Gradual and stable therapeutic effects are preferable, as they are associated with lower risk of misuse.
- Timing of SMO initiation: SMO should only be introduced once the patient has achieved opioid stabilization and sustained abstinence from heroin.
- Hierarchical pharmacological approach: MMT should be considered a prerequisite before initiating SMO therapy.

Limitations: This study has several limitations. Its retrospective design precludes any conclusions about causality, and the small sample size limits statistical power and generalizability. However, given the severity of the clinical population studied—individuals with chronic polysubstance use—these preliminary findings are clinically meaningful. They provide an empirical foundation upon which future prospective studies can be built, ideally with larger samples, standardized protocols, and longer follow-up periods.

## 5. Conclusions

Long-term treatment with SMO appears to be effective in reducing relapse risk among individuals with co-occurring AUD and HUD who are receiving opioid agonist therapy. Despite concerns regarding its potential for misuse, SMO—when co-administered with methadone in a controlled clinical setting—was associated with favorable outcomes and no significant adverse effects in our sample. The stabilizing effects of opioid maintenance treatment may help mitigate the abuse liability of SMO, supporting its role as a viable therapeutic option in patients with poly-addictions. These findings underscore the need for prospective, controlled studies specifically designed to evaluate the safety and efficacy of SMO in populations with polysubstance use.

## Figures and Tables

**Table 1:** Baseline demographics.

	MM/SMO-Maintained	MM/SMO-Detoxified		*p*
	Med (Q1–Q3)	Med (Q1–Q3)	z *	
Age	37 (28–51)	42 (33–51)	0.63	0.528
	N (%)	N (%)	χ^2^	
Sex, female	13 (61.9)	9 (42.9)	1.52	0.217
Education, <8 years	13 (61.9)	15 (71.4)	0.43	0.513
Marital status, with partner	14 (66.7)	13 (61.9)	0.10	0.747
Employment, blue collar	14 (66.7)	13 (61.9)	0.15	0.929
Income, adequate	18 (85.7)	19 (90.5)	0.23	0.634
Living situation, not alone	17 (81.0)	15 (71.4)	0.52	0.469

* Mann–Whitney U test.

**Table 2:** CGI codes.

Illness Severity	Efficacy Index	Therapeutic Effect
0. Not assessed	0. Not assessed	1. Marked/No side effect
1. Normal, not at all ill	1. Very much improved	2. Marked/Do not interfere with patient functioning
2. Borderline mentally ill	2. Much improved	3. Marked/Interferes with patient functioning
3. Mildly ill	3. Minimally improved	4. Marked/Outweighs therapeutic effect
4. Moderately ill	4. No change	5. Moderate/No side effect
5. Markedly ill	5. Minimally worse	6. Moderate/Do not interfere with patient functioning
6. Severely ill	6. Much worse	7. Moderate/Interferes with patient functioning
		8. Moderate/Outweighs therapeutic effect
		9. Minimal/No side effect
		10. Minimal/Do not interfere with patient functioning
		11. Minimal/Interferes with patient functioning
		12. Minimal/Outweighs therapeutic effect
		13. Unchanged or worse/No side effect
		14. Unchanged or worse/Do not interfere with patient functioning
		15. Unchanged or worse/Interferes with patient functioning
		16. Unchanged or worse/Outweighs therapeutic effect

**Table 3:** Association between sex, age, baseline severity of illness, and type of treatment received with alcohol relapsing behavior in 42 HUD alcoholics treated with MM after SMO alcohol detoxification or with methadone/SMO maintenance.

	N	B	Exp(B)	95% CI	*p*
Male sex (N = 20) Female sex (N = 22)	22 20	−0.41	1.00 0.96	0.02–1.94	0.935
Age	42	−0.02	0.97	0.93–1.01	0.204
Baseline severity of illness	42	0.44	1.56	0.72–2.40	0.296
MM-SMO detoxified MM-SMO maintained	21 21	−1.89	1.00 0.15	0.03–0.27	0.001

χ^2^ = 14.44 df 4 *p* = 0.006.

**Table 4:** Endpoint CGI indexes comparison between MM/SMO-detoxified vs. MM/SMO-maintained patients.

	MM/SMO- Detoxified	MM/SMO- Maintained	z *	*p*
	Med (Q1–Q3)	Med (Q1–Q3)		
Endpoint CGI illness severity	5.00 (4.00–6.00)	4.00 (3.00–4.50)	−2.42	0.015
Endpoint CGI efficacy index	4.00 (3.00–4.00)	1.00 (1.00–2.00)	−4.58	0.000
Endpoint CGI therapeutic effect	13.00 (9.00–14.00)	2.00 (1.00–5.50)	−4.40	0.000

* Mann–Whitney U test.

## Data Availability

We can share our research data on request.

## Abbreviations

The following abbreviations are used in this manuscript:

AUD	Alcohol Use Disorder
CGI	Clinical Global Impressions
CNS	Central Nervous System
DSM	Diagnostic Statistical Manual
GABA	Gamma-aminobutyric Acid
GHB	Gamma-Hydroxybutyric Acid
HUD	Heroin Use Disorder
MM	Methadone Maintenance
MMT	Methadone Maintenance Treatment
OUD	Opioid Use Disorder
RCT	Randomized Controlled Trial
SMO	Sodium Oxybate
SUD	Substance Use Disorder
